# MiR-218-5p Affects Subcutaneous Adipogenesis by Targeting *ACSL1*, a Novel Candidate for Pig Fat Deposition

**DOI:** 10.3390/genes13020260

**Published:** 2022-01-28

**Authors:** Baosen Shan, Mengting Yan, Kai Yang, Weimin Lin, Jiayu Yan, Shengjuan Wei, Wei Wei, Jie Chen, Lifan Zhang

**Affiliations:** College of Animal Science and Technology, Nanjing Agricultural University, Nanjing 210095, China; shanbaosen@126.com (B.S.); 2018105035@njau.edu.cn (M.Y.); 2019105037@njau.edu.cn (K.Y.); 2018205002@njau.edu.cn (W.L.); 15118305@njau.edu.cn (J.Y.); sjwei@njau.edu.cn (S.W.); wei-wei-4213@njau.edu.cn (W.W.); jiechen@njau.edu.cn (J.C.)

**Keywords:** miRNAs, miR-218-5p, *ACSL1*, subcutaneous fat deposition, pigs

## Abstract

As a centre enzyme in fatty acid activation, acyl-CoA synthetase long-chain family member 1 (ACSL1) plays an important role in body lipid homeostasis. However, the functions of *ACSL1* in the subcutaneous adipogenesis of pigs are largely unknown. In the present study, we found that the expression of *ACSL1* significantly increased during the process of porcine preadipocyte differentiation. Moreover, silencing of *ACSL1* in preadipocytes decreased levels of triglyceride and adipogenic-related markers, including *FABP4*, *APOE*, and *FASN* (*p* < 0.01), and simultaneously increased levels of lipolytic-related markers, such as *ATGL* and *HSL* (*p* < 0.05). Conversely, overexpression of *ACSL1* in preadipocytes increased levels of triglyceride and *FABP4*, *APOE*, and *FASN* (*p* < 0.01), and reduced levels of *ATGL* and *HSL* (*p* < 0.05). Luciferase reporter assays revealed that *ACSL1* is a target of miR-218-5p, which can reduce the mRNA and protein levels of *ACSL1* by directly binding the 3′ untranslated region of *ACSL1*. Furthermore, miR-218-5p has an inhibition role in porcine preadipocyte differentiation by suppressing *ACSL1* expression. Taken together, these data provide insights into the mechanism of the miR-218-5p/*ACSL1* axis in regulating subcutaneous fat deposition of pigs.

## 1. Introduction

Subcutaneous fat is one of the main factors affecting pig growth and meat quality. For obese pig breeds, such as most Chinese native pig breeds, excessive subcutaneous fat deposition becomes a major issue for the pig industry as it greatly increases the cost of feeding and production. But for lean pig breeds, appropriate subcutaneous fat deposition is favourable for meat quality because of its positive correlation with intramuscular fat content in muscle [1]. Studying the molecular mechanism of subcutaneous fat adipogenesis could provide the potential gene targets for improvement of the production efficiency and meat quality in the pig industry. In general, adipogenesis undergoes a series of complex physiological processes, in which multipotent stem cells derived from fat are gradually converted into preadipocytes and differentiated mature adipocytes [2]. Preadipocyte differentiation is known to be regulated by multiple key genes, including peroxisome proliferator activated receptor gamma (*PPARγ*) and CCAAT enhancer binding protein alpha (*C*/*EBPα*) [3]. However, many other potential genes, such as forkhead box O1 (*FOXO1*) [4], PR/SET domain 16 (*PRDM16*) [5], and stearoyl-CoA desaturase (*SCD*) [6], also play an important role in the adipogenic differentiation of porcine preadipocytes. Therefore, identifying the genes related to adipogenesis and resolving their action will help us to understand the formation mechanism of subcutaneous fat deposition, thus providing a basis for molecular breeding of subcutaneous fat of pigs.

As an essential material for adipogenesis, fatty acid can be metabolised in two different ways: decomposition into acetyl-CoA or merging into triacylglycerol and phospholipid, which contain a common starting step of fatty acid activation [7]. This step requires the participation of the acyl-CoA synthetase long-chain (ACSL) family, which can transform long-chain fatty acids to fatty acyl-CoA esters. In the ACSL family, ACSL1 is a lipid droplet-associated protein and has been shown to play a critical role in lipid metabolism in humans [8]. For example, overexpression of *ACSL1* increases triglyceride contents in the liver [9], while knockout of *ACSL1* reduces the conversion of oleate to triacylglycerol [10]. In brief, for gestational age-derived adipocytes, the expression of *ACSL1* is associated with lipid levels [11]. Furthermore, our previous transcriptome data show that the expression of porcine *ACSL1* is dramatically increased during the subcutaneous preadipocyte differentiation [12], suggesting that *ACSL1* might be an important functional gene in subcutaneous adipogenesis of pigs.

MicroRNAs (miRNAs) are a length of 20–24 nucleotide non-coding RNAs, which are involved in various cellular regulatory processes, including adipocyte formation, by silencing the expression of their target genes [13,14]. Previously, many miRNAs have been found to affect lipid metabolism by targeting *ACSL1* in different species. For example, miR-205 influences abnormal lipid metabolism in hepatoma cells [15], miR-181a affects the biosynthesis of milk fat in bovine mammary epithelial cells [16], and miR-34-5p enhances hepatic lipid content in chickens [17]. However, the role of the *ACSL1* and *ACSL1*-miRNA axis in the adipogenesis of pigs is still largely unknown. Therefore, in this study, the mechanism of the *ACSL1*-miRNA axis in subcutaneous adipogenesis was investigated, providing a new clue for comprehending the subcutaneous fat adipogenesis of pigs.

## 2. Materials and Methods

### 2.1. Animals

The seven-day-old Chinese Erhualian piglets were purchased from Changzhou Erhualian Pig Production Cooperation (Changzhou, Jiangsu, China). All animal experiments, including the preadipocytes collected, were in accordance with the guidelines and regulations of the Animal Ethics Committee at Nanjing Agricultural University (STYK(su)2011-0036).

### 2.2. Isolation, Primary Culture, and Differentiation of Porcine Preadipocytes

The isolation of preadipocytes was described in our previous report [12]. In brief, subcutaneous adipose tissue in the back of each pig was cut and digested with 1 mg/mL collagenase type I (Sigma-Aldrich, St. Louis, MO, USA) at 37 °C in a water bath for 1.5 h, followed by a filtration using a 200 μm nylon mesh. The preadipocytes were collected by centrifuging the filtrated solution at 800 g for 10 min. Cells were cultured in a growth medium (Dulbecco’s modified Eagle’s medium F-12 (DMEM-F12) containing 10% fetal bovine serum (FBS) and 1% penicillin-streptomycin) under 37 °C with an atmosphere of 5% CO_2_. After the growth of the preadipocytes was completely confluent, the induction medium (DMEM-F12 containing 10% FBS, 1 mol/L dexamethasone, 5 μg/mL insulin, 0.1 mmol/L isobutyl-1-methylxanthine, and 1% penicillin-streptomycin) was replaced to induce cell differentiation for 2 d. Then, cells were cultured using a maintenance medium (growth medium supplemented with 5 μg/mL insulin) for 6 d, which was changed every other day.

### 2.3. Quantitative Real-Time RT-PCR (qRT-PCR)

Total RNA of cell samples was extracted by using TRIzol kit (Invitrogen, Carlsbad, CA, USA) according to the manufacturer’s protocol. The cDNA synthesis for mRNA and miRNA was performed using PrimeScript RT reagent kit (Takara, Dalian, China) and miRNA RT-PCR kit (TransGen, Beijing, China), respectively, according to the manufacturer’s instructions. The qRT-PCR was performed on a StepOne Plus system (Thermo Fisher Scientific, Waltham, MA, USA) using the AceQ qPCR SYBR Green Master Mix (Vazyme, Nanjing, China). The primers for qRT-PCR were designed by using Premier 5.0 and listed in Supplementary Table S1. Ribosomal protein lateral stalk subunit P0(*RPLP0*) and *U6* small nuclear RNA were used as endogenous controls for genes and miRNAs, respectively. Assays were performed in triplicate. The 2^−ΔΔCT^ method was used to calculate the expression differences [18].

### 2.4. Transfection of miRNA, siRNA, and Plasmid

The small interfering RNA (siRNA) sequences of *ACSL1* were designed and are listed in Supplementary Table S2. All siRNAs, miRNAs, and plasmids were transfected using Lipofectamine 2000 (Invitrogen, Carlsbad, CA, USA) according to the manufacturer’s protocol. The freshly isolated cells proliferated for 96 h were seeded in 6-well plates for transfection at 80–90% confluence. After 48 h of transfection, cells were collected for qRT-PCR and Western blotting analysis.

### 2.5. Determination of Oil Red O Staining and Triglyceride Contents of Adipocytes

Adipocytes were gently washed thrice with phosphate buffered solution (PBS). After adding 4% paraformaldehyde, the cells were incubated at 37 °C for 30 min, followed by washing thrice with PBS. Next, the cells were added with Oil Red O solution for 30 min at room temperature and then were washed thrice with PBS. Finally, these cells were observed using an inverted microscope (Leica, Wetzlar, Germany). The triglyceride contents of adipocytes were measured at the absorbance value of 510 nm using a full-wavelength microplate reader (Thermo Fisher Scientific, Waltham, MA, USA), and assays were performed in triplicate.

### 2.6. Western Blotting Analysis

Total protein of cells was extracted using cell lysis buffer. The concentration of total protein was determined using a BCA protein concentration kit (Beyotime, Shanghai, China) according to the manufacturer’s protocol. Next, the protein was electrophoresed on a 12% SDS-PAGE gel with 135 V for 90 min. Then, the protein was transferred to a polyvinylidene fluoride membrane (Millipore, Billerica, MA, USA) with 90 V for 100 min. The membrane was blocked for 1.5 h with 5% bovine serum albumin (BSA) and incubated with a diluted monoclonal anti-ACSL1 antibody (1:1000) or anti-GAPDH antibody (1:10,000) at 4 °C overnight, followed by a secondary goat anti-rabbit antibody (1:10,000) for 60 min. Protein bands were visualised using ECL luminescent solutions, and the signal intensity was quantified using the Image J software.

### 2.7. Construction of ACSL1 Overexpression Plasmid

The coding sequence (CDS) sequence of *ACSL1* was cloned using a cloning primer as follows: the upstream primer contained the *NheI* endonuclease site sequence, 5′-CGGCTAGCCGATGATGCAAGCCCACGAGCT-3′; the downstream primer contained *KpnI* endonuclease site sequence, 5′-GGGGTACCCCTTAGACTTTGACGGTGGAAT-3′. The cloned product was ligated into empty pcDNA3.1+ vector (Invitrogen, Carlsbad, CA, USA), transformed using DH5α competent cells (Vazyme, Nanjing, China), and purified by an endotoxin-free plasmid extraction kit (Omega, Doraville, GA, USA).

### 2.8. Construction of Luciferase Reporter Vector

The 3′ untranslated region (UTR) sequence of porcine *ACSL1* (GenBank: GBZA01000015.1) was obtained from the NCBI database. Three bioinformatics tools, including TargetScan (http://www.targetscan.org, accessed on 15 March 2018), miRbase (http://www.mirbase.org, accessed on 7 June 2018), and miRWalk (http://mirwalk.umm.uni-heidelberg.de, accessed on 18 October 2018), were used to predict the targeted miRNAs with potential combination with the 3′UTR of *ACSL1*. The 3′UTR sequence containing the miRNA binding site was synthesised and subcloned into the pmirGLO vector (Vazyme, Nanjing, China). The wild-type (wt) and mutant (mut) vector of the 3′UTR of *ACSL1* were constructed, respectively. The oligonucleotide sequences of mimics and inhibitor of miRNA are shown in Supplementary Table S2.

### 2.9. Dual-Luciferase Activity Assay

The HEK 293T/17 cells proliferated for 48 h were transiently transfected. After 48 h, the culture medium in a 12-well plate was discarded, and cells were washed thrice with PBS. According to the instructions of the dual-luciferase assay system kit (Promega, San Luis Obispo, CA, USA), a total of 200 μL of cell lysate was added, and the plate was shaken for 15 min. Next, the lysate from each well was collected in a 1.5 mL centrifuge tube and was centrifuged at 5500× *g* for 1 min. The supernatant was pipetted into a new 1.5 mL centrifuge tube. A total of 4 μL lysate was mixed with 20 μL of LARII, and the firefly fluorescence value was detected on a fluorescence detector. Then, 20 μL of 1 × stop solution was added to determine the renilla fluorescence value. The value of the last fluorescence activity was the firefly fluorescence value/renilla fluorescence value.

### 2.10. Statistical Analysis

Statistical analysis of the data was performed using SPSS v26.0 software. Student’s *t*-test was used for comparisons between two groups, while one-way analysis of variance (ANOVA) was used for evaluating the difference among three or more groups, followed by Duncan’s multiple comparison test. Data are presented as the mean ± standard error (SEM). The *p* values less than 0.05 and 0.01 were considered as statistically significant and highly significant, respectively.

## 3. Results

### 3.1. Changes of Preadipocyte Differentiation and the Expression of ACSL1 in Differentiated Preadipocytes

**Figure 1:**
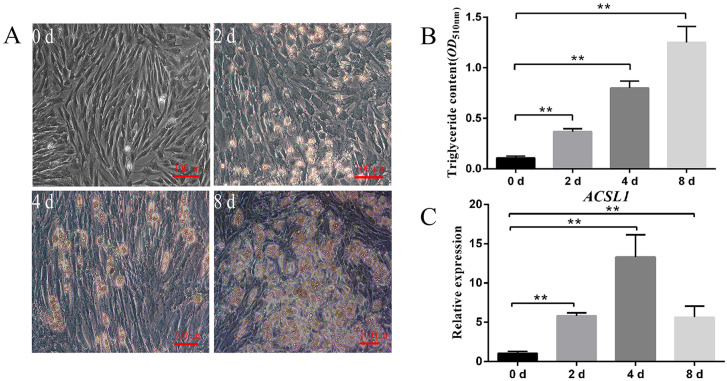
Differentiation of porcine preadipocytes and the expression pattern of *ACSL1* in differentiated preadipocytes. Morphological (**A**) and triglyceride content (**B**) changes of preadipocytes at d 0, d 2, d 4, and d 8. (**C**): mRNA levels of *ACSL1* at different differentiation stages. D 0, d 2, d 4, and d 8: 0 h, 48 h, 96 h, and 192 h after adipogenic induction. Data are shown as mean ± SEM. *n* = 3 per group. **: *p <* 0.01.

During the preadipocyte differentiation process, lipid droplets were observed to become larger with the increase in the triglyceride contents (Figure 1A,B). Meanwhile, the expression of *ACSL1* also increased, accompanying cell differentiation. On the fourth day, the levels of *ACSL1* reached a peak (Figure 1C).

### 3.2. Effect of siRNA Interference of ACSL1 on Differentiation of Preadipocytes

**Figure 2:**
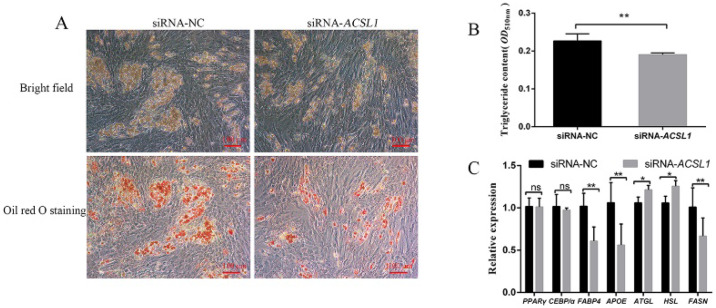
Effect of siRNA interference with *ACSL1* on preadipocyte differentiation. Interfering with *ACSL1* inhibits preadipocyte differentiation phenotype (**A**) and triglyceride content (**B**) at d 8. (**C**) Effect of interference with *ACSL1* on preadipocyte differentiation-related genes at d 2. D 2 and d 8: 48 h and 192 h after adipogenic induction. Data are shown as mean ± SEM. *n* = 3 per group. *: *p* < 0.05, **: *p* < 0.01, *ns*: *p* < 0.05.

To explore the role of *ACSL1* in preadipocyte differentiation, we examined the effect of siRNA-*ACSL1* on the differentiation of preadipocytes. Among the three siRNAs targeting *ACSL1* (Figure S1A,B), siRNA-321 had the highest interference efficiency and was used for further study. On the eighth day of preadipocyte differentiation, the number of lipid droplets in siRNA-*ACSL1*-treated cells was less than that in siRNA-NC-treated cells (negative control; Figure 2A). Correspondingly, the triglyceride contents of siRNA-*ACSL1*-treated cells were lower than that in siRNA-NC-treated cells (*p* < 0.01) (Figure 2B). Moreover, interfering *ACSL1* expression reduced the expression levels of adipogenic-related fatty acid binding protein 4 (*FABP4*), apolipoprotein E (*APOE*), and fatty acid synthase (*FASN*) genes (*p* < 0.01) and simultaneously increased the levels of lipolytic-related adipose triglyceride lipase (*ATGL*) and hormone-sensitive lipase (*HSL*) genes (*p* < 0.05), with no effect on the expression levels of the early differentiation marker *PPARγ* and *C*/*EBPα* genes (*p* < 0.05) (Figure 2C).

### 3.3. Effect of ACSL1 Overexpression on Differentiation of Preadipocytes

**Figure 3:**
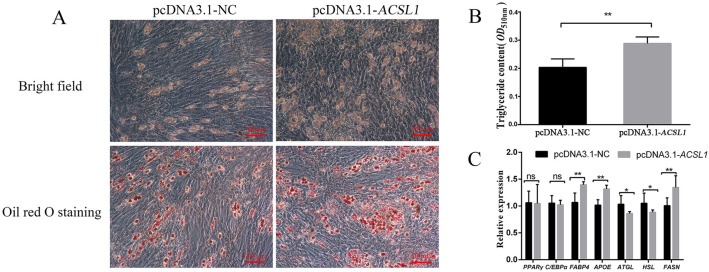
Effect of overexpression of *ACSL1* on preadipocyte differentiation. Effect of overexpression of *ACSL1* on preadipocyte differentiation phenotype (**A**) and triglyceride content (**B**) at d 8. (**C**) Effect of overexpression of *ACSL1* on preadipocyte-differentiation-related genes at d 2. D 2 and d 8: 48 h and 192 h after adipogenic induction. Data are shown as mean ± SEM. *n* = 3 per group. *: *p* < 0.05, **: *p* < 0.01, *ns*: *p* < 0.05.

To further verify the role of *ACSL1* in preadipocyte differentiation, the *ACSL1* overexpression vector was constructed. The results showed the expression of *ACSL1* in preadipocytes treated with pcDNA3.1-*ACSL1* was dramatically higher than that in cells treated with pcDNA3.1-NC (Figure S2A,B), indicating that the overexpression vector of *ACSL1* could be used for further experiment. After 8 d of induction culture, the number of lipid droplets with pcDNA3.1-*ACSL1* treatment was greater than that in the control groups. Moreover, the triglyceride contents in the pcDNA3.1-*ACSL1*-treated cells were higher than those in the control groups (*p* < 0.01) (Figure 3A,B). Overexpression of *ACSL1* resulted in an increase in the expression levels of the lipogenic-related *FABP4*, *APOE*, and *FASN* genes (*p* < 0.01) (Figure 3C) and a decrease in the levels of lipolytic-related *ATGL* and *HSL* genes (*p* < 0.05), with no influence on the expression levels of *PPARγ* and *C*/*EBPα* (*p* < 0.05).

### 3.4. MiR-218-5p Targeting the 3′UTR of ACSL1

**Figure 4:**
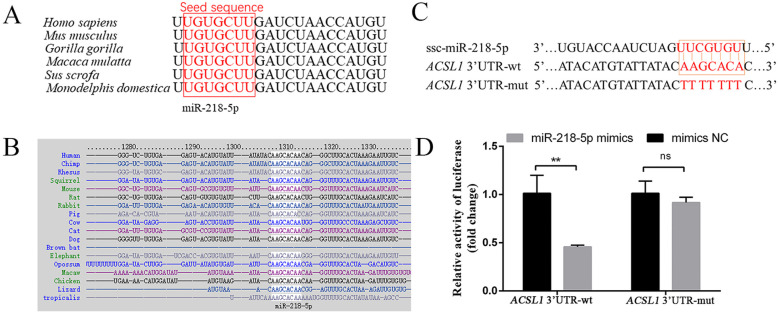
MiR-218-5p targets the 3′UTR of *ACSL1*. (**A**) The conserved mature sequence of miR-218-5p in different species. (**B**) Conservative analysis of the binding site of miR-218-5p to the 3′UTR of *ACSL1* in different species. (**C**) The wild-type (wt) and mutant (mut) 3′UTR fluorescent reporter vector plasmids of *ACSL1* were aligned with miR-218-5p. (**D**) Luciferase activity of pmirGLO-*ACSL1* 3′UTR-wt and pmirGLO-*ACSL1* 3′UTR-mut co-transfected with miR-218-5p mimics for 24 h. Data in D are represented as mean ± SEM of six experimental replicates. **: *p* < 0.01, *ns*: *p* < 0.05.

Bioinformatics analysis revealed that hsa-miR-218-5p is a potential miRNA for targeting the human *ACSL1* 3′UTR (Supplementary Figure S3). Moreover, the mature sequence of miR-218-5p and its binding site for *ACSL1* was highly conserved among different mammals (Figure 4A,B). To investigate whether porcine miR-218-5p could directly bind the predicted site on the porcine *ACSL1* 3′UTR, the luciferase reporter vectors containing the binding site of the wild-type *ACSL1* 3′UTR (*ACSL1* 3′UTR-wt) and mutated binding site sequences (*ACSL1* 3′UTR-mut) were constructed further (Figure 4C). The relative luciferase activity was decreased after co-transfection of miR-218-5p mimics and the wild-type vector (*p* < 0.01), while no difference was observed after co-transfection of miR-218-5p mimics and the mutant vector (*p* < 0.05) (Figure 4D), indicating that miR-218-5p could directly target *ACSL1* by binding its 3′UTR.

### 3.5. MiR-218-5p Inhibits Preadipocyte Differentiation and ACSL1 Expression

**Figure 5:**
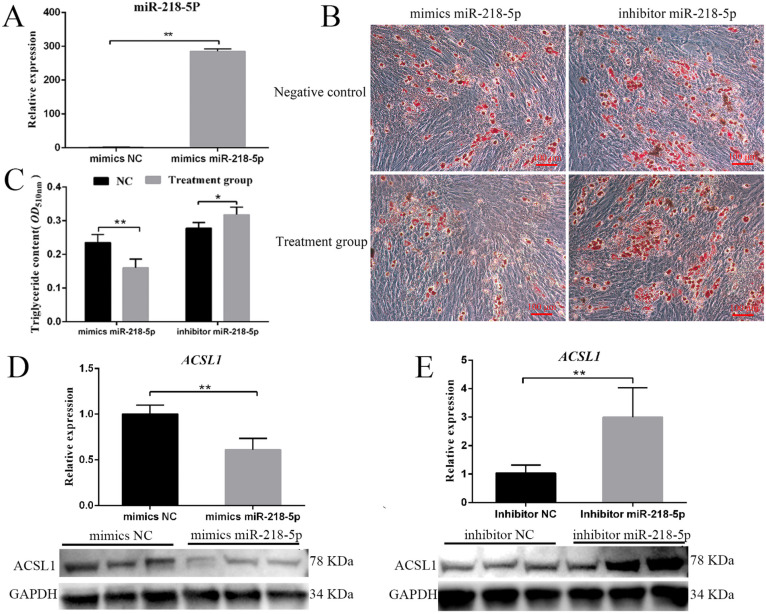
MiR-218-5p inhibits preadipocyte differentiation. (**A**) MiR-218-5p overexpression in preadipocytes at d 2. (**B**) Effect of miR-218-5p overexpression and inhibitor on preadipocyte differentiation at d 8. (**C**) Effect of miR-218-5p overexpression and inhibitor on triglyceride content at d 8. (**D**) Effect of miR-218-5p overexpression on *ACSL1* at mRNA and protein levels at d 2. (**E**) Effect of miR-218-5p inhibitor on *ACSL1* at mRNA and protein levels at d 2. Treatment group: adipocytes treated with miR-218-5p mimics (left) or inhibitor (right). D 2 and d 8: 48 h and 192 h after adipogenic induction. Data are shown as mean ± SEM. *n* = 3 per group, *: *p* < 0.05, **: *p* < 0.01.

Our results showed that the overexpression of miR-218-5p decreased triglyceride contents (*p* < 0.01) and the number of lipid droplets, whereas the inhibition of miR-218-5p increased triglyceride contents (*p* < 0.05) and the number of lipid droplets (Figure 5A–C), indicating that miR-218-5p inhibits preadipocyte differentiation. Furthermore, the overexpression of miR-218-5p reduced both mRNA and protein levels of *ACSL1* (*p* < 0.01), whereas the knock-down of miR-218-5p increased both mRNA and protein levels of *ACSL1* (*p* < 0.01) (Figure 5D,E).

### 3.6. MiR-218-5p Regulates Preadipocyte Differentiation by Targeting ACSL1

**Figure 6:**
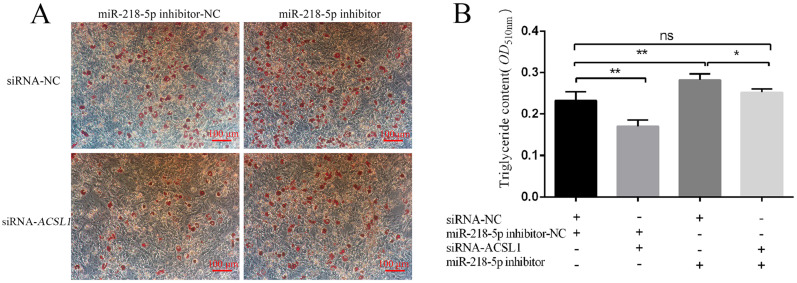
MiR-218-5p targeting *ACSL1* affects preadipocyte differentiation. Effect of siRNA-*ACSL1*, miR-218-5p inhibitor, and co-transfection of both on preadipocyte differentiation at d 8 was determined by Oil Red O staining (**A**) and triglyceride content detection (**B**). D 8: 192 h after adipogenic induction. “+” and “−”: the materials (siRNA-NC, miR-218-5p inhibitor-NC, siRNA-*ACSL1*, and miR-218-5p inhibitor) were transfected (+) or not transfected (−) into preadipocytes. Data are shown as mean ± SEM. *n* = 3 per group, *: *p* < 0.05, **: *p* < 0.01, *ns*: *p* < 0.05.

To confirm whether miR-218-5p affects preadipocyte differentiation via targeting *ACSL1*, preadipocytes were cultured for adipogenic differentiation after transfecting siRNA-*ACSL1*. The data showed that the number of lipid droplets and triglyceride contents were reduced compared with siRNA-NC treatment cells (*p* < 0.01 for triglyceride contents) (Figure 6A,B). Conversely, the number of lipid droplets and triglyceride contents were increased in cells treated with miR-218-5p inhibitor compared with the cells with miR-218-5p inhibitor-NC (*p* < 0.01 for triglyceride contents) (Figure 6A,B). Moreover, no difference was observed for the number of lipid droplets and triglyceride contents in cells treated with both the siRNA-*ACSL1* and miR-218-5p inhibitor compared with those in cells treated with siRNA-NC and miR-218-5p inhibitor-NC (*p* < 0.05). Inhibition of both the *ACSL1* and miR-218-5p attenuated the miR-218-5p-inhibitor-induced lipid deposition (*p* < 0.05) (Figure 6A,B).

## 4. Discussion

Previously, the expression levels of *ACSL1* in subcutaneous fat tissue and its allele frequencies of SNPs between obese and lean pig breeds showed a significant difference [19], indicating that this gene has a function in the subcutaneous fat deposition of pigs. As this gene is a key enzyme involved in fatty acid metabolism, we speculated that *ACSL1* might play a role in the subcutaneous fat deposition of pigs. Therefore, by using a preadipocyte differentiation model, we further tested the relationship between *ACSL1* and the subcutaneous adipogenesis of pigs.

Our results showed that the expression levels of *ACSL1* progressively increased during the process of porcine preadipocyte differentiation (Figure 1C), implying that *ACSL1* might be involved in the regulation of subcutaneous adipogenesis. Moreover, the data from interference and overexpression of *ACSL1* indicated that *ACSL1* could promote preadipocyte differentiation and the expression of the lipogenic-related *FABP4*, *APOE*, and *FASN* genes [20,21,22] and simultaneously suppress the expression of lipolytic-related *ATGL* and *HSL* genes (Figure 2C and Figure 3C) [23,24], strongly supporting that *ACSL1* is a positive player in the subcutaneous adipogenesis of pigs. These results are consistent with another study, which revealed the expression of *ACSL1* significantly increased, and its knockdown reduced the triglyceride contents in human mesenchymal stem-cell-derived adipocytes [9]. However, the role of *ACSL1* in preadipocyte differentiation in different species is still controversial. For example, *ACSL1* overexpression significantly increased the contents of diglyceride rather than triglyceride levels compared with the control group in sheep adipocytes [25], and *ACSL1* knockdown did not show significant changes in the uptake of long-chain fatty acids, lipid droplet size, and glycerol levels compared with the control group in mouse 3T3-L1 cells [26]. Here our data provide new evidence that *ACSL1* could positively regulate the porcine preadipocyte differentiation.

Additionally, our data showed that such positive roles of *ACSL1* in preadipocyte differentiation is not achieved by affecting the expression of early key differentiation-related markers of *PPARγ* and *C*/*EBPα* genes, but by regulating lipolysis-related markers of *ATGL* and *HSL* genes, thus affecting the final adipogenic phenotype of adipocytes (Figure 2A–C and Figure 3A–C). In adipocytes differentiated from mesenchymal stem cells, *ACSL1* knockdown also fails to show any significant effect on the expression of *PPARγ* and *C*/*EBPα* [9]. In other studies, *ACSL1* had been found to influence triglyceride contents of liver cells via the *PPARγ* pathway [27], and *ACSL1* overexpression increased the levels of *PPARγ* and the lipid droplets of bovine adipocytes [28], which is not fully consistent with our data. Therefore, the relationship of *ACSL1* and *PPARγ* pathways in different cells from different species needs further investigation.

MiR-218-5p is a highly conserved miRNA in mammals, which has been considered as a tumour suppressor by inhibiting cell proliferation, migration, growth, and metastasis of many cancers, including breast cancer, prostate cancer, and cervical cancer [29,30,31]. Additionally, many studies also found that miR-218-5p can regulate skin and hair development [32,33]. However, little is known about the function of *ACSL1* in the adipogenesis of agricultural animals. In our research, we found that miR-218-5p can directly target the 3′ UTR region of the porcine *ACSL1* (Figure 4C,D). The levels of *ACSL1* and the triglyceride phenotype were dramatically decreased and increased when miR-218-5p was overexpressed and inhibited (Figure 5B–E), respectively, demonstrating that miR-218-5p can inhibit the expression of *ACSL1* and preadipocyte differentiation. Furthermore, our data confirmed that miR-218-5p inhibits preadipocyte differentiation by specifically reducing *ACSL1* expression (Figure 6A,B). Taken together, these results give us a new clue for the role of miR-218-5p in the adipogenesis of pigs.

## 5. Conclusions

This study revealed that *ACSL1* had a promoting effect on the subcutaneous preadipocyte differentiation of pigs, and miR-218-5p could inhibit preadipocyte differentiation by targeting *ACSL1*. Therefore, altering the levels of miR-218-5p can be considered a valuable way to regulate subcutaneous adipogenesis in pigs.

## Data Availability

Not applicable.

## Supplementary Materials

The following are available online at https://www.mdpi.com/article/10.3390/genes13020260/s1, Figure S1: Effect of RNA interference of *ACSL1*. (A) The expression of *ACSL1* detected by qRT-PCR in porcine preadipocytes treated with siRNA-NC or siRNA-*ACSL1*. (B) The expression of *ACSL1* detected by Western blotting in porcine preadipocytes treated with siRNA-NC or siRNA-*ACSL1*. Data are shown as mean ± SEM. *n* = 3 per group. *: *p* < 0.05, **: *p* < 0.01, Figure S2: Effect of *ACSL1* overexpression. (A) The expression of *ACSL1* detected by qRT-PCR in porcine preadipocytes treated with pcDNA3.1 or pcDNA3.1-*ACSL1*. (B) The expression of *ACSL1* detected by Western blotting in porcine preadipocytes treated with pcDNA3.1 or pcDNA3.1-*ACSL1*. Data are shown as mean ± SEM. *n* = 3 per group. *: *p* < 0.05, **: *p* < 0.01, Figure S3: The binding site prediction between hsa-miR-218-5p and human *ACSL1*, Table S1: List of the primer sequences, Table S2: List of siRNA and oligonucleotide information.
