# Supplementary figures and images for: MiR-218-5p Affects Subcutaneous Adipogenesis by Targeting ACSL1, a Novel Candidate for Pig Fat Deposition
Source: Genes (Basel). 2022 Jan 28;13(2):260. doi: 10.3390/genes13020260 (PMC8871969; doi:10.3390/genes13020260)

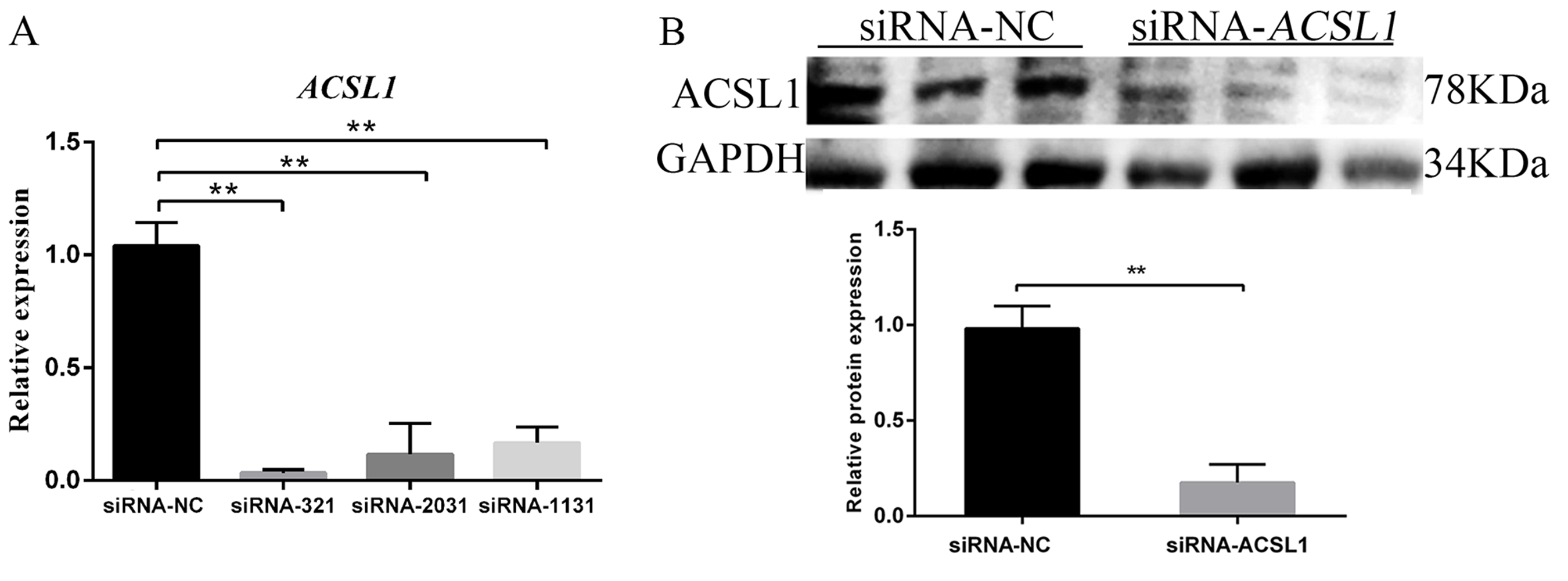

Supplement: Supplementary file 1 [file genes-13-00260-s001.zip › Supplementary/Supplementary Figure S1 Effect of RNA interference of ACSL1 gene.tif]

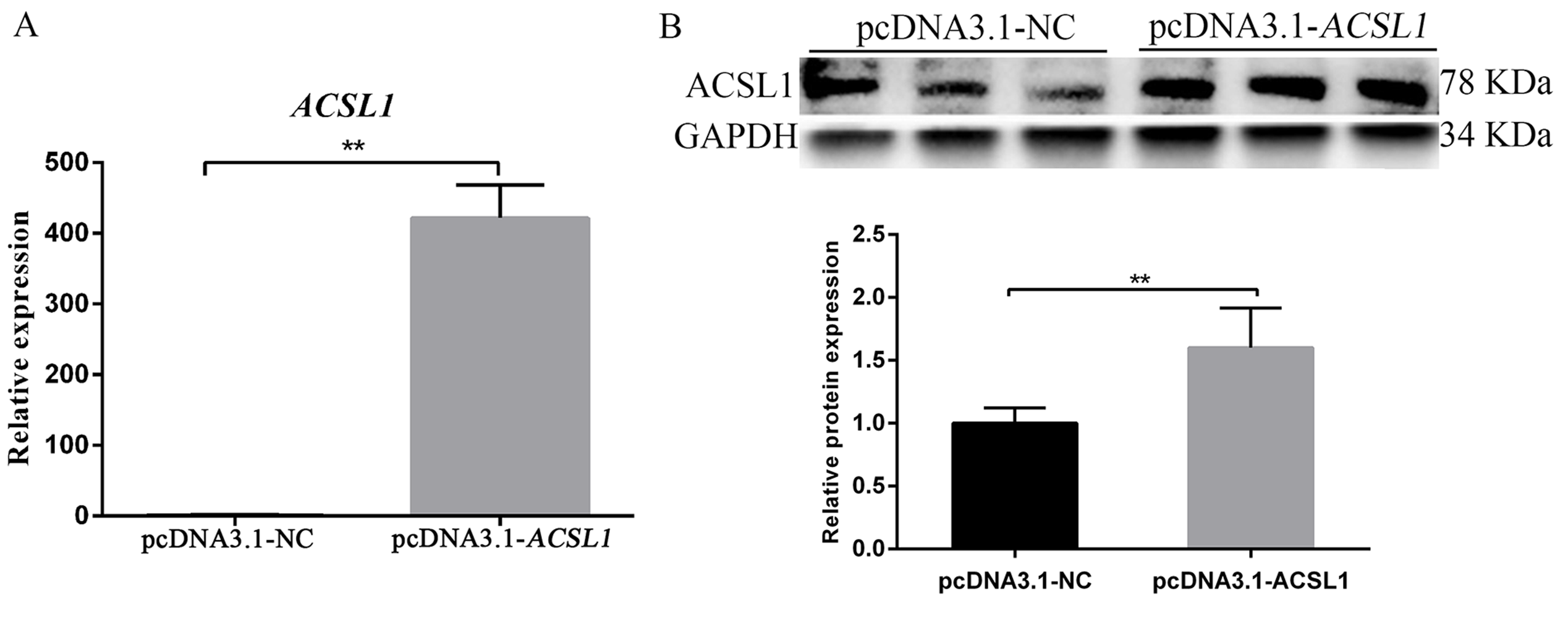

Supplement: Supplementary file 1 [file genes-13-00260-s001.zip › Supplementary/Supplementary Figure S2 Effect of ACSL1 overexpression.tif]

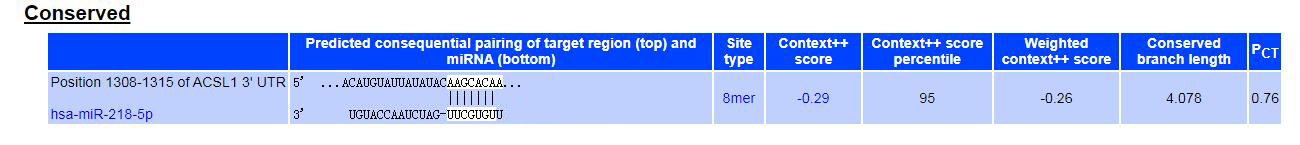

Supplement: Supplementary file 1 [file genes-13-00260-s001.zip › Supplementary/Supplementary Figure S3 The binding site prediction between hsa-miR-218-5p and human ACSL1.tif]
